# Structural and Functional Characterization of Lipoxygenases from Diatoms by Bioinformatics and Modelling Studies

**DOI:** 10.3390/biom14030276

**Published:** 2024-02-25

**Authors:** Deborah Giordano, Simone Bonora, Ilenia D’Orsi, Domenico D’Alelio, Angelo Facchiano

**Affiliations:** 1 Institute of Food Sciences, National Research Council, via Roma 64, 83100 Avellino, Italy; deborah.giordano@isa.cnr.it (D.G.); sbonora@unisa.it (S.B.); ilenia.dorsi1990@gmail.com (I.D.); 2 Department of Integrative Marine Ecology, Stazione Zoologica Anton Dohrn, Villa Comunale, 80121 Naples, Italy; domenico.dalelio@szn.it; 3 National Biodiversity Future Center (NBFC), Piazza Marina 61, 90133 Palermo, Italy

**Keywords:** lipoxygenase, diatoms, protein modelling, sequence clustering, docking analysis

## Abstract

Lipoxygenases make several biological functions in cells, based on the products of the catalyzed reactions. In diatoms, microalgae ubiquitous in aquatic ecosystems, lipoxygenases have been noted for the oxygenation of fatty acids with the production of oxylipins, which are involved in many physiological and pathological processes in marine organisms. The interest in diatoms’ lipoxygenases and oxylipins has increased due to their possible biotechnological applications, ranging from ecology to medicine. We investigated using bioinformatics and molecular docking tools the lipoxygenases of diatoms and the possible interaction with substrates. A large-scale analysis of sequence resources allowed us to retrieve 45 sequences of lipoxygenases from diatoms. We compared and analyzed the sequences by multiple alignments and phylogenetic trees, suggesting the possible clustering in phylogenetic groups. Then, we modelled the 3D structure of representative enzymes from the different groups and investigated in detail the structural and functional properties by docking simulations with possible substrates. The results allowed us to propose a classification of the lipoxygenases from diatoms based on their sequence features, which may be reflected in specific structural differences and possible substrate specificity.

## 1. Introduction

Diatoms, microalgae ubiquitous in aquatic ecosystems, from freshwater to marine environments, are single-celled organisms with silica shells and multiple biotechnological purposes [1]. These microscopic algae are integral to aquatic food webs, supporting a myriad of organisms, generating oxygen, shaping food webs, and sequestering carbon [2]. The biological diversity and ecological adaptability of diatoms underscore their profound impact on aquatic ecosystems. The main driver of such an impact is the production of chemical compounds with extra-cellular functions, such as the oxylipins produced by lipoxygenase biochemistry.

Diatom-derived oxylipins are end products of well-characterized enzymatic pathways activated after cell wounding, starting from the lipolytic release of fatty acids (FAs) from complex lipids and proceeding through oxygenation of FAs by lipoxygenases (LOX) [3,4,5,6,7,8]. The ecological significance of diatom lipoxygenases and oxylipins extends beyond cellular metabolism, shaping community structure and functioning, highlighting their intricate role in maintaining the balance and resilience of aquatic environments. Oxylipins have multifaceted functions in aquatic ecosystems, mediating prey–prey and prey–predator signaling processes and communication within plankton food webs [9,10,11]. By producing oxylipins, diatoms influence the food searching behavior [12], reproductive fitness [13,14] and gene expression [15,16] of crustacean copepods, the dominant zooplankton assemblage that provides a strong trophic link between primary producers and secondary consumers (fish, jellyfish) [17]. In addition, there is interest in the possible biotechnological applications of diatom oxylipins for exploiting, as some studies suggested, their anti-cancer, anti-bacterial, anti-fungal and anti-parasitic activities (for a review see [18].

Besides their role in aquatic environments as info-chemicals, allelochemicals, deterrents against grazers and mediators influencing carbon recycling, recent studies additionally demonstrated that oxylipins exert anti-inflammatory activity, can promote tissue regeneration, alleviate pain [19], and regulate angiogenesis, blood pressure, blood coagulation, and blood vessel permeability [20]. Moreover, due to their capability to promote the activation of apoptotic pathways in cancer cells, to regulate adhesion, migration and proliferation, oxylipins can also exert antiproliferative activity on human cancer cells [21,22,23].

The LOX enzymes are responsible for the oxidation of polinsatured fatty acids to oxylipins. This reaction takes place in the LOX catalytic U-shaped pocket in the presence of an iron ion as a cofactor and a water molecule. The cofactor is located in a central position, at the base of the “U pocket”, and is usually coordinated by five residues: three His, one Asn and one Ile. The iron cofactor extracts a hydrogen atom to the nearest carbon of the substrate, at two positions upstream or downstream of the oxidation of the double bond, according to the substrate orientation in the pocket [24]. Generally, if the substrate polar head is located externally to the active site, the oxidation takes place two positions after the carbon atom nearest to the iron; otherwise, if the tail is oriented to the external side, then it takes place two positions before, based on the substrate double bond placed at a minor distance from the oxygen channel present in the protein. According to the position of the double bond oxidized, it is possible to have different LOX types also for the same substrate [25]. The LOX 3D structure is generally well preserved among bacteria, plants and mammals [26], however, the amino acid composition of the U-shaped cavity jointly with the conformational change that could be induced by substrates or inhibitors, plays a crucial role in LOX specificity [27]. A detailed explanation about the enzyme substrate recognition mechanism and about the enzyme activity can be found in ref. [24] and [27] and in their figures. Moreover, a visual representation of the interaction occurring among substrate cofactor and coordination residues is visible in Supplementary Figure S1.

In this study, we analyzed a wide range of biomolecular data relating to diatom LOXs available from public repositories, from which we modelled the fine-scale 3D structure of different categories of these enzymes present in the biochemical machinery of diatoms belonging to different phylogenetic groups. The detailed structural and functional analysis suggested a classification and a characterization of the putative LOX from diatoms, giving a hypothesis on their substrate specificity.

## 2. Materials and Methods

### 2.1. LOX Sequences: Detection, Analysis and Clustering Procedure

Starting from the sequences annotated as LOX in Uniprot and NCBI, it was possible to select two reference sequences: the LOX from *Fragilariopsis cylindrus* CCMP1102 (accession number NCBI: OEU18547.1); and the LOX from *Pseudo-nitzschia arenysensis* (accession number NCBI: QWC64745.1). These sequences have been used as NCBI BLAST [28,29] queries to search for other putative LOX protein sequences in diatoms by exploiting different databases of both protein sequences and transcripts. In the details, blastp was used to search the two queries versus the Non-redundant protein sequence (nr) database and the Transcriptome Shotgun Assembly proteins (tsa nr) database in the taxon of diatoms (taxid: 2836); while tblasn was exploited for searching the queries versus the Nucleotide collection (nr/nt) database, the Whole-genome shotgun contigs (wgs) database, and the Transcriptome Shotgun Assembly (TSA), again, narrowing the search to the taxid: 2836. The hypothetical LOX nucleotide sequences obtained have been translated by the Expasy Translate Tool [30].

Similarly to the approach used in previous studies of our lab [31], sequences collected have been standardized by performing multiple sequence alignments using Clustal Omega [32] by a visual inspection, focusing on their length and the presence along the sequence of the LOX domain, and by manually cutting and removing each different domain detected. Domain detection has been performed by exploiting the ScanProsite tool [33] and Search InterProScan [34]. The standardized sequences obtained have been clustered by the construction of several phylogenetic trees performed by MEGA tool 6.06 [35], using Maximum Likelihood algorithm, a bootstrap of 100, the Jones–Taylor–Thornton (JTT) substitution model, all sites for gaps data treatment, and the Subtree-Pruning-Regrafting-Fast (SPR level 3) for the ML heuristic method. Group and subgroup division were performed on the base of the obtained branches, grouped by a bootstrap value major of 95 and according to the mutation occurring at the fourth cofactor coordination residue.

To search conserved regions, motifs or patterns along the sequences of each group detected after the clustering procedure, MEME suite [36] was used.

### 2.2. 3D Models Construction and Docking Analysis

For each group detected, a representative protein sequence was chosen for further structural and docking analyses. Their simple modeling by AlphaFold2 was not satisfactory because the procedure did not generate the active conformation of the enzyme, with iron ion and related coordinating side chains correctly folded, functional water molecule, and appropriate access of the substrate to the active site. Therefore, we adopted a strategy to model LOXs of *Fragilariopsis cylindrus* and *Pseudo-nitzschia arenysensis* with the correct active conformation features, and then used these models as a template for modelling the other LOX sequences. Similar procedures are routinely applied in our laboratory for several protein models’ construction and investigation (for a recent example, see [37]). In detail, the crystallographic structure of the allene oxide synthase-lipoxygenase protein from *Plexaura homomalla* (PDB code: 3FG1) and of the lipoxygenase 3 from *Glycine max* (PDB code: 1HU9) have been used as templates for modelling the LOX sequences isolated from *Fragilariopsis cylindrus* and *Pseudo-nitzschia arenysensis*, respectively. A first multiple modelling step was performed using SWISS-MODEL [38]; subsequently, the best model obtained for each sequence was used, jointly with the PDB structures mentioned before, as template for the building of the final models employing Modeller10.2 [39]. Each model obtained is subjected to a validation process that considers the stereochemical model quality, its total energy, its geometrical score and the most suitable conformation (correct position of the cofactor coordination atoms, open conformation of the active site, etc.). Conformational energy, backbone and side-chain stereo-chemical characteristics, and suitability with known properties were evaluated using the web servers ProSA-web [40], QMEAN [41] and PROCHECK [42].

The best models obtained have been exploited for the model refinement of the Alpha Fold models obtained for the other sequences selected. Therefore, the two models of LOX from *Fragilariopsis cylindrus* and *Pseudo-nitzschia arenysensis* were in turn used as templates by Modeller10.2 to refine the models of the other representative putative LOX sequences built by the ColabFold tool of ChimeraX v1.5 [43]. Details about the modelled sequences and their templates are reported in Table 1.

This refinement procedure allowed us to construct all models in active conformation, in presence of an iron ion as a cofactor, with a correct orientation of its coordination residues, and of a functional water molecule; the latter added exploiting the 3D structure of a lipoxygenase in a complex with arachidonate from *Plexaura homomalla* (PDB code: 4QWT).

Once created the 3D structures in the active conformation of all the sequences selected, focused docking analyses with flexible and rigid protocols were performed using AutoDock and AutoDockTools 4.2.6. [44], testing as substrates: eicosapentaenoic acid (EPA) (PubChem CID: 446284), hexadeca-6Z,9Z,12Z-trienoic acid (HTrA) (PubChem CID: 52921838), hexadeca-6Z,9Z,12Z,15-tetraenoic acid (HTtA) (PubChem CID: 11957734), docosa-4Z,7Z,10Z,13Z,16Z,19Z-esenoic acid (DHA) (PubChem CID: 445580), 9,12-hexa-decadienoic acid (PubChem CID: 5282787), and 4,7,10,13,16,19,22,25-octacosaoctaenoic acid (28: 8n–3) (PubChem CID: 54316623). Ligands were made flexible in all simulations. To allow the accommodation of the substrates, flexibility in some amino acid side chains was adopted when some pockets, due to the particular hindrance of some amino acids that composed them, did not allow substrate binding by a simple rigid methodology of docking. Details about the docking parameters adopted are reported in Supplementary Table S1. As positive control of the procedure, a re-docking was simulated exploiting the crystallographic structure of the complex made up of the LOX from *P. homomalla* and the arachidonic acid (AA) (PDB code: 4QWT). The binding energy values we report refer to the free energy of binding of ligands to receptors estimated by the AutoDock 4.2 force field.

## 3. Results

### 3.1. Putative LOX Sequences Detection, Analysis and Classification

From sequence database searching, we found that the only protein sequences annotated as lipoxygenase in diatoms are the ones from *Pseudo-nitzschia arenysensis*, *Fragilariopsis Cylindrus*, and *Nitzschia inconspicua.* Although the first two have the same annotation and a similar length, 704 residues for LOX from *P. arenysensis* and 742 for LOX from *F. cylindrus*, from their alignment it is possible to notice that they are dissimilar; it is possible to detect a similarity of 21% and in only 20% of sequence coverage. Concerning the LOX of *N. incospicua*, this sequence seems to be wrongly annotated due to the absence of a domain attributable to LOX, and from its modelling the structure obtained is not comparable to a typical LOX structure. Thus, starting from these two sequences, different transcriptome and protein sequence databases have been searched (see methods for details), leading to the detection of about a hundred sequences, most of them annotated as hypothetical proteins. Sequence fragments, or sequences not preserving at least three of the five cofactor coordination residues, and sequences without a putative LOX domain have been removed from the list.

**Figure 1:**
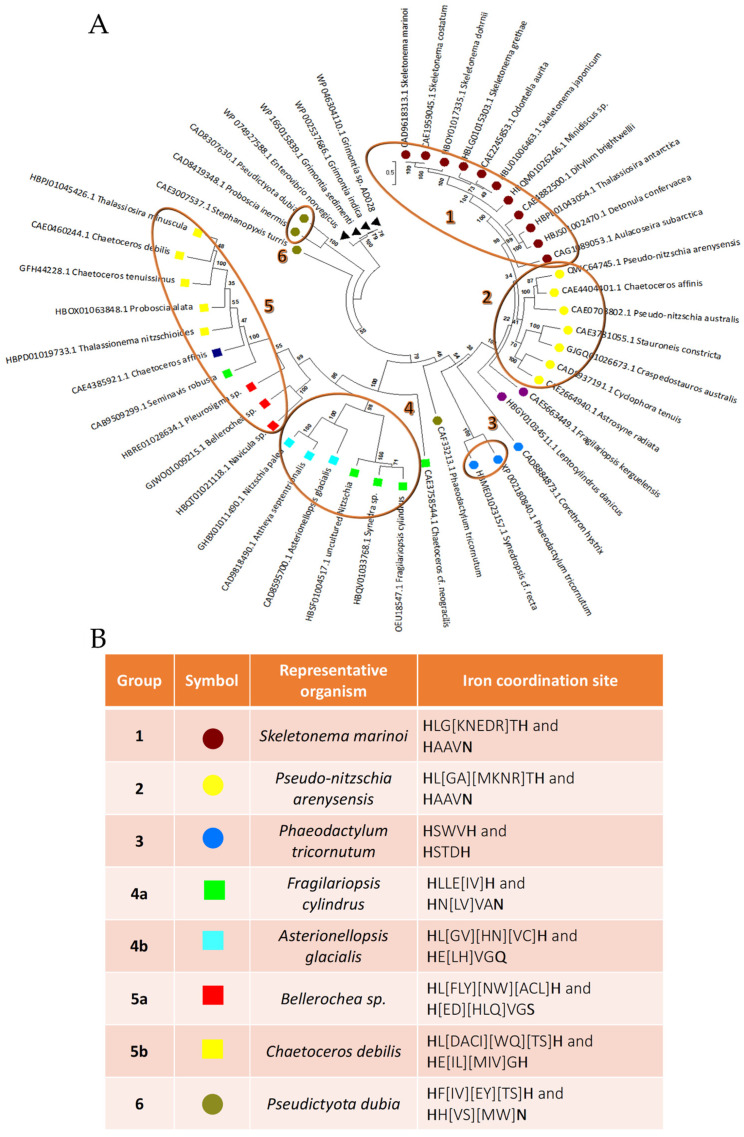
Phylogenetic analysis. (**A**) Phylogenetic tree representing the clustering of the LOX sequences analyzed. Highlighted by number and circle are the six different major groups detected. Marked by a circle are the sequences similar to LOX from *P. arenysensis*, and by a square the ones more similar to *F. cylindrus*. In group 4, under the same branch grouping them with a bootstrap value of 100, subgroups can be distinguished as 4a (marked by green squares) and 4b (by cyan squares). Similarly, in group 5, subgroup 5a (red square under the same branch) is distinguishable from the rest (group 5b under the branch with a bootstrap value of 100) due to the same bootstrap split; LOX sequence from *Pleurosigma* sp. seems to be an intermediate form between the two subgroups. Black triangles indicate sequences exploited for the root of the tree. The colors of the labels refer to specific iron coordination residue compositions visible in the symbol section reported in panel B. (**B**) Reference sequence representative of each group detected by the phylogenetic three and their relative cofactor coordination pattern. Each group has as a symbol: a circle or a square, according to the major similarity with LOX from *P. arenysensis* or *F. cylindrus*, respectively. Different colors refer to specific labelled sequences grouped by a circle in the tree. Residues directly involved in the cofactor coordination are highlighted in bold.

By this procedure, it was possible to collect 45 possible LOXs from diatoms, found to be similar to the LOX from *P. arenysensis* and *F. Cylindrus* (Supplementary Table S2). Thus, starting from an optimized multiple alignment of all the sequences selected, a clustering analysis was performed by the construction of a phylogenetic tree. As expected, the results obtained highlight that the sequences found can be divided into two major groups: one collecting sequence, similar to LOX from *P. arenysensis*, and the other one from *F. Cylindrus.* However, the tree analysis suggests that the 45 sequences can be further divided into at least six groups (bootstrap value on branch grouping them > 95) (Figure 1A), among which, groups four and five can be in turn divided into two subgroups according to the mutation of the fourth cofactor coordination residue and the presence of two different branches grouping sequences with a bootstrap value equal to 100. Groups 1, 2, and 3 include most of the sequences similar to LOX from *P. arenysensis,* although a few other sequences are on the opposite side of the tree (group 6).

All groups, except for the first and the second, differ either in the distance along the sequence of the second couple of cofactor coordination residues, or in the fourth coordination residue that could be histidine, glutamine, or serine, instead of asparagine.

In detail, group 3 presents a deletion between the first two histidine residues of coordination, and the substitution of the asparagine of coordination with another histidine. Group 4 presents, in both its subgroups, an insertion between the third and the fourth coordination residue; however, in subgroup 4b this residue is substituted by glutamine. Group 5 presents an insertion between the third and the fourth coordination residue too, but its asparagine of iron coordination is substituted by serine and histidine in 70% of the sequences composing subgroups 5a (yellow squares in the tree) and all the sequences composing subgroup 5b plus LOX from *Pleurosigma* sp. (red squares in the tree), respectively. Group 6 seems to preserve, similar to groups 1 and 2, the same cofactor coordination residues; however, the position of the third residue represents a tricky point due to the presence of two histidine residues one after the other that do not allow to detect which one is involved in the coordination, on the basis of sequence alignment. A detailed description of the coordination pattern is displayed in Figure 1B (last column).

From the analysis of motifs/patterns present along the sequences, it was possible to detect the reason why groups 1 and 2 emerge as separated. Even if both groups preserve the same coordination residues, they differ in the presence of a specific pattern in group 2 that is absent in group 1 and vice versa (Supplementary Figure S2).

### 3.2. 3D Models and Docking Analysis

For each group, we chose a representative protein sequence that we modelled in an active conformation, in the presence of both water and a cofactor. For the first group, we selected LOX from *S. marinoi*; for the second, LOX from *P. arenysensis*; for the third, LOX from *P. tricornutu*, LOX from *F. cylindrus*, and *A. glacialis* for groups 4a and 4b, respectively; LOX from *Bellerochea* sp. for group 5a and LOX from *C. debilis* for 5b; and LOX from *P. dubia* for group 6 (Figure 1B).

From the models obtained it was possible to notice that the deletion that occurred in group 3 does not seem to affect the correct orientation of the coordination residues (Supplementary Figure S3C). In a similar way, in groups 4 and 5, despite the insertion, the asparagine or the residue which replaces it, i.e., glutamine, histidine or serine, is able to reach the correct position to coordinate the cofactor (Supplementary Figure S3D–G). Moreover, the model obtained for the sequence representative of group 6 highlighted that between the two consecutive residues of histidine found in the correct position for the cofactor coordination, the functional role seems to be carried out by the first (Supplementary Figure S3H). After checking the quality of the models obtained and the compatibility of their structures with an active conformation, docking analyses were performed with EPA, the main fatty acid present in diatoms, or other suitable substrates selected from the literature, to analyze the LOX type to which each putative LOX selected belongs to.

In the quality of positive control of the docking approach applied, the first analysis performed regarded the re-docking of the LOX from *P. homomalla* and the arachidonic acid; complex is already available in the PDB. Our results completely reproduced the crystallographic data and as reported in the literature [27], indicating LOX8-type activity. As in the crystallographic structure, the AA placed itself with its polar head outside the catalytic pocket, and the cofactor interacts with the C10 of the substrate. Due to the presence of a Gly in position 427 that defines the top of the putative O2 channel, the oxygen necessary for the reaction is free to flow in this upper side of the channel [45,46] and to attack the C8 of the AA, leading to the generation of 8R products.

Similarly, we have analyzed docking results obtained for the other representative putative LOXs with the suitable substrates selected. Results have shown that the pocket composition determines different interactions for the same substrate and influences the substrate selectivity of the enzyme (Table 2).

### 3.3. Docking Analyses for Sequences Similar to LOX from P. arenysensis

**Figure 2:**
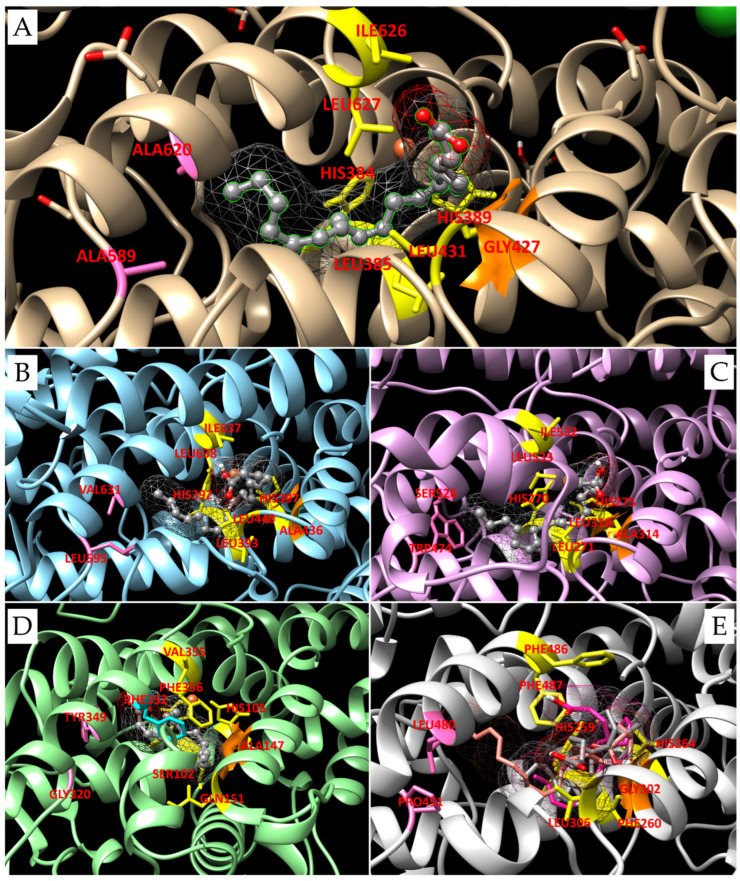
Active site pocket composition in LOX from *P. homomalla* (**A**), *P. arenysensis* (**B**), *S. marinoi* (**C**), *P. tricornutum* (**D**) and *P. dubia* (**E**). The ligands are represented with a grey ball and stick; the residues responsible for their orientation in the pocket are highlighted in yellow sticks; while the ones composing the deepest portion of the catalytic pocket are highlighted in pink; and glycine and alanine, that open or close, respectively, the upper side of the oxygen channel are represented in orange surfaces. In (**D**), represented in cyan sticks, Phe352 probably partially closes the deepest region of the active site; while in (**E**), beyond the octacosaoctaenoic acid, represented in grey sticks, DHA and EPA are shown in pink and magenta, respectively.

For LOX from *P. arenysensis*, docking with EPA has shown an interaction similar to that of the positive control. For LOX from *P. homomalla,* six conserved amino acids, i.e., the first coordination couple (His384/389), three Leu (385/431/627) and one Ile (626), are strategically located to allow the attack of the iron ion on the C10 of the substrate [27] (Figure 2A); all these residues are conserved also in the LOX from *P. arenysensis*.

According to our results, the EPA carbon that interacts with the iron ion is carbon 10. However, in this case Ala (in position 436, in place of the corresponding Gly427 in LOX from *P. homomalla*), determines the occlusion of the upper side of the oxygen channel, driving the O2 deeper into the cavity; this causes an O2 reaction with the carbon 12 of the substrates; therefore, this enzyme shows a LOX12 behavior. Moreover, the binding energy and constant of inhibition are comparable between the LOX of *P. arenysensis* and the control (−7.40 Kcal/mol with a Ki of 3.74 µM for LOX from *P. homomalla* and −7.59 Kcal/mol with Ki equal to 2.75 µM for LOX from *P. arenysensis*). Interestingly, the LOX from the *P. homomalla* active site is characterized by a deep cavity due to the presence at its terminal part of two Alanine residues in position 589 and 620. The LOX from *P. arenysensis* does not preserve these residues, showing in their place Leu595 and Val631; however, this cavity reduction is not enough to impede EPA binding (Figure 2B).

In the case of LOX from *S. marinoi*, the active site preserves the six conserved amino acids involved in the correct location of the substrate, even if Ile626 is replaced by a leucine, but changes the composition of the terminal part of the pocket with Ala589/620 replaced by Ser526 and Trp474. The presence of Trp in place of Ala determines a shallower cavity, while the Ser is suitable for better interaction with the polar head of the substrates. From our simulations is possible to detect that EPA is not able to interact using its C10 with the iron ion, whose attack is limited to C7 (Figure 2C). This conformation results in a higher binding energy (mean binding energy equal to −5.46 Kcal/mol, lowest binding energy equal to −7.04 Kcal/mol) than the ones detected for the positive control (mean binding energy equal to −6.95 Kcal/mol, lowest binding energy equal to −7.40 Kcal/mol). The reduction of the pocket volume, due to the Trip presence, probably limits the deepening of the hydrocarbon tail. For what concerns the smaller substrates tested, HtrA and HTtA, both bind the active site with their carboxyl head located inside the pocket and interact with the iron ion by their C11. Also in this case, the upper side of the oxygen channel is locked by the presence of the Ala314, so the enzyme behavior can be associated with a LOX9 for all three substrates tested. Generally, looking at the binding energies, we can see that they are comparable for EPA and HTrA and higher for HTtA, revealing a preference of this LOX for EPA and HTrA.

Hypothetical LOX from *P. tricornutum* presents many differences from the ones described until now. Of the six residues implicated in the orientation of the substrate, it preserves only the two histidines, while Leu385/431/627 is replaced by Ser102, Gln151 and Phe356, and Ile626 is replaced by Val355. Moreover, Ala589/620 is replaced by Gly320 and Tyr349, and Gly427 by Ala147. All these modifications influence the substrate binding, which seems to be able to move deeper inside the external part of the pocket, and Phe356 seems to drive the substrate fold inside it. Moreover, the substitution of Thr623 with Phe352 partially closes the deepest region of the active site, while the deletion between the two His of coordination and the replacement of Leu385 with Ser102 create more space in this area. Docking simulations performed with EPA for this hypothetical LOX did not highlight any noteworthy result; simulations performed with 9,12-hexa-decadienoic acid show a binding energy comparable to the one obtained for *S. marinoi* LOX and suggest a possible interaction of the iron ion with the C8, resulting in a possible LOX12 because of the closure of the upper side of the oxygen channel. O2 is probably able to reach only the last part of the ligand thanks to its particular orientation. The substrate seems capable of binding the pocket in an unconventional manner, with the polar head and the tail oriented toward Val355 and Gln151, respectively (Figure 2D). Among the representative sequences selected, the last one, more similar to the one from *P. arenysensis*, is the hypothetical LOX from *P. dubia*. This sequence preserves the coordination residues and the canonical number of residues among them; however, it presents a higher variability in the composition of this region. Many substitutions occur; in particular, Pro451 and Leu480 completely close the end of the pocket, replacing Ala589/620; among the six residues necessary for locking the substrate conformation suitable for the iron attack, only His384/389 and Leu431 (at positions 259/264 and 306, respectively, in this sequence) are preserved, the others are replaced by three phenylalanine residues. The Gly427 (Gly302 in LOX from *P. dubia*) opening the higher portion of the oxygen channel is preserved. Docking simulations have been performed with different substrates: EPA, DHA and octacosaoctaenoic acid. All the ligands are positioned with their hydrocarbon tail inside the deepest portion of the pocket and their carboxyl head outside (Figure 2E). However, each substrate binds the iron ion at different positions of their hydrocarbon chain: EPA with its C10, DHA with C6 or C9 that are equally distant from the iron, even if looking at the substrate conformation the interaction with the C9 seems the most probable, and octacosaoctaenoic acid with C18, resulting in a LOX8, LOX4/7 or LOX16, respectively. Looking at the binding energy, it is possible to detect a preference for DHA and octacosaoctaenoic acid, which show the lowest binding energies and Ki values comparable to the ones detected for AA and EPA interaction with the LOX from *P. homomalla* and *P. arenysensis*, respectively.

### 3.4. Docking Analyses for Sequences Similar to LOX from F. cylindrus

**Figure 3:**
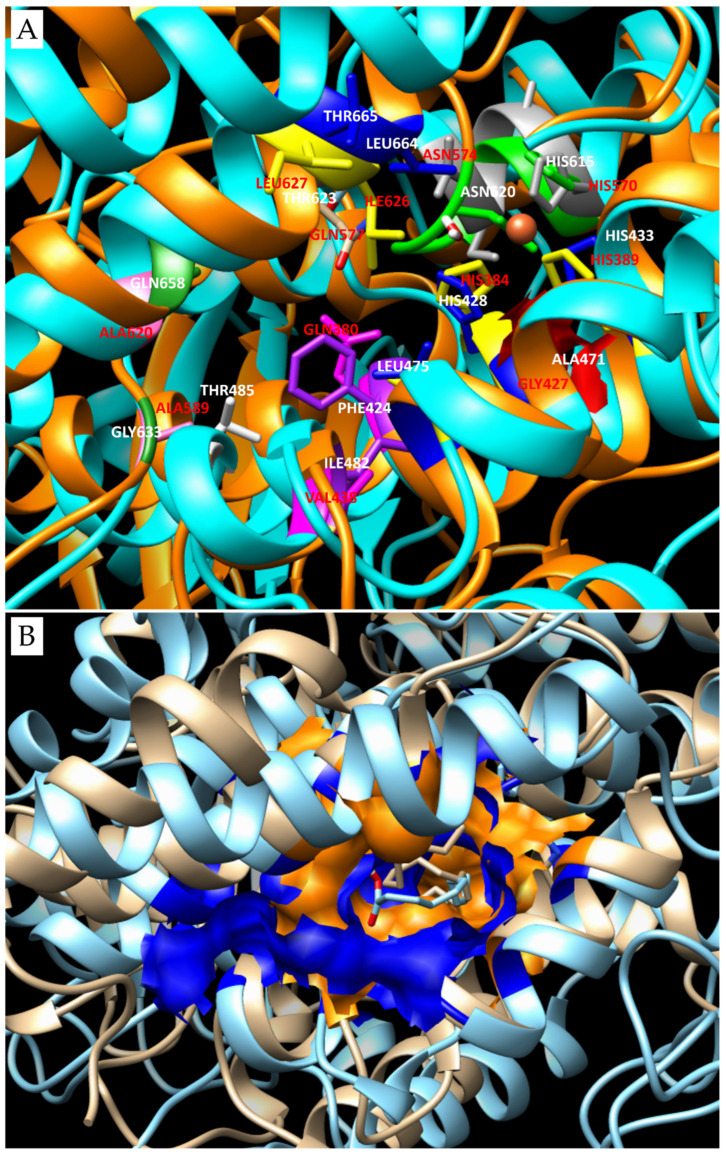
Active site pocket comparison between LOX from *F. cylindrus* and LOX from *P. homomalla* or/LOX from *P. arenysensis.* (**A**) Comparison of the aminoacidic composition of the active site pockets present in LOX from *P. homomalla* and *F. cylindrus.* The superimposition procedure was adopted, but the two sequences are completely different, and this has an impact on the two structures whose overlap generates a high RMSD value, i.e., 4.615 Å. In orange ribbons are the LOX from *F. cylindrus* with relevant residues composing the active site pocket highlighted in blue, violet, green, white, dark green and cyan sticks, all indicated by white labels; while the Ala471 locking the O2 channel is represented with a red surface. The LOX from *P. homomalla* is represented in orange cartoons with its relevant residues highlighted in yellow, pink, magenta, orange, grey and beige, all indicated by red labels. (**B**) Shape comparison of the active site pocket of LOX from *P. arenysensis*, with a blue surface in a structure represented in cyan ribbons; and LOX from *F. cylindrus*, with an orange surface in a structure represented in beige ribbons. In this case, too, the superimposition of the structures has a high RMSD value, i.e., 8.866 Å, due to very strong difference between the protein sequences. The different active site compositions determine differences in terms of pocket shape and substrate binding.

Docking results related to LOX from *Fragilariopsis cylindrus* are a clear example, as the different orientations of the substrates determine different LOX types. The structure preserves four of the six amino acids necessary for the correct substrate orientation in the pocket, with Ile626 and Leu627 replaced by Leu664 and Thr665, respectively. Ala471 locks the upper side of the O2 channel. Gln658 and Gly633 replace the Ala589/620 forming the back of the pocket. Moreover, in this region, Gln380 and Val438 present in LOX from *P. homomalla* are replaced by Phe424 and Ile482, which shrink the area. In particular, Phe424 opposite to Thr485, jointly with Gln658, further reduces the access to the back of the pocket. The second coordination couple, i.e., His615 and Asn620, preserves its correct conformation for the interaction with the iron ion. However, the insertion between the couple of one more residue, in addition to the canonical three, results in the break of the alpha helix hosting them and determines a protrusion in the consequently formed coil inside the active site pocket. Moreover, the presence of Thr623, which replaces the Gln577 of LOX from *P. homomalla*, residue preserved also in LOX from *P. arenysensis* and *S. marinoi*, together with the already mentioned Thr665 in place of Leu627, and Phe424 in place of Gln380, creates a larger area before the access to the deeper portion of the cavity. This region is comfortable for the substrate docking, which could make novel interactions with the portion embedded between the coordination residue couple His/Asn, including also the backbone of the novel inserted Ala619. Thus, this insertion, jointly with the substitution that occurred in the deeper portion of the active site, results in a slight modification of the active site pocket shape that becomes larger but shorter (Figure 3A,B) and is suitable to host the substrate in different conformations.

**Figure 4:**
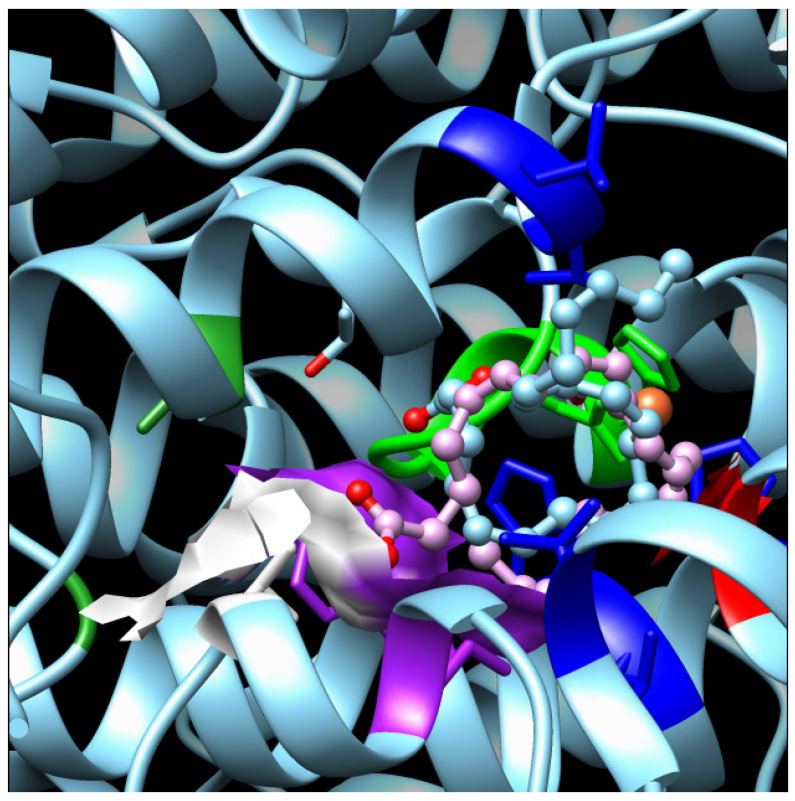
Different orientations of the EPA substrate in the LOX from the *F. cylindrus* active site. The enzyme is represented by a cyan carton; Phe424 and Ile482, which narrowed the entrance in the deepest portion of the cavity, are highlighted in a violet surface together with Thr485 in white surface; in the blue sticks are the residues responsible for the correct orientation of the substrate in the pocket; in green is the region between the second couple of iron coordination residues: His615 and Asn620 are highlighted with a stick. In cyan is Thr623, which creates further space in the cavity. EPA is represented with pink/cyan balls and sticks.

Our docking results show that EPA can bind the active site in a double conformation, for each of which its carboxyl head is located either inside the larger area at the entrance of the deeper portion of the pocket or in the first part of the latter. The EPA tail, instead, is oriented either in the external part of the pocket or folded, exploiting the external part of the pocket at the larger area in the center. In particular, when the polar head of EPA is oriented in the center of the pocket, the iron interacts with the carbon 10 of the substrate, and vice versa; when it is oriented in the deeper portion of the pocket, the carbon nearest to iron, is C13. This means that in the first case, the enzyme behavior is typical of a LOX8 in the second of a LOX15 (Figure 4). No preference seems to be present for one or the other conformation because both the binding energies are quite comparable, as are the inhibition constants.

A clustering analysis collected the sequences of the group represented by *F. cylindrus* LOX sequence near another one, for which LOX from *Asterionellopsis glacialis* has been chosen, but under the same clade. A hypothetical LOX from *A. glacialis* preserves four residues of the six necessary for the ligand orientation. In particular, Ser696 and Thr697 substitute Ile626 and Leu627, respectively; the deepest portion of the pocket is composed of Thr673 and Leu690 in place of Ala589/620, and Gln380 and Val438 (detectable in LOX from *P. homomalla*) are replaced by Thr459 and Phe517, respectively; while the upper side of the O2 channel is closed by Ala507 instead of Gly427. The presence of Gln660 in the replacement of the Asn of coordination seems to not affect the iron binding; however, in this case also, the insertion among the last couple of coordination residues breaks the alpha helix. Having no information about the enzyme products in this organism, for this LOX, three different substrates with different lengths have been chosen to detect a possible preference. All the substrates bind the enzyme with their polar head outside and their tail inside the pocket; however, the EPA bound the iron with its C6. Therefore, the oxidation occurs in position 9, DHA with its C12; thus, the enzyme behavior is typical of a LOX14, instead of the octacosaoctaenoic acid with its C18, so the oxidation happens in position 20.

In group 5, two main clades can be distinguished, so we decided to analyze them both, focusing our attention primarily on the hypothetical LOX sequence from *Bellerochea* sp., which seems drastically split from the other sequences. Among these, we selected hypothetical LOX from *Chaetoceros debilis* that appears to be the most recent.

Hypothetical LOX from *Bellerochea* sp. seems to be characterized by the deepest pocket. Moreover, Ala620 is preserved while Ala589 is substituted by Thr471, but the residues preceding it, Ala-Pro in *P. homomalla* LOX, are replaced by Ala and Gly in *Bellerochea* sp. Moreover, residues from 579 to 583 (Asp-His-Tyr-Gly-Phe), in alpha helix conformation in *P. homomalla,* are replaced by a coil (Leu-Thr-Ser-Pro), which creates more space in the last portion of the pocket and reduces the central part of it. That is because the iron coordination residues His453 and Ser458 are found just before these residues, and the insertion among this couple generates, as in the previously mentioned LOX, a break in the helix and a kinked conformation of the coil that invades the active site and warps it. Among the residues of coordination of the substrate, His279/284 and Leu280/327 (corresponding to His384/389 and Leu385/431 in LOX from *P. homomalla*, respectively) are preserved; Leu494 and Thr495 replace Ile626 and Leu627. Also in this case, the oxygen channel is closed by the replacement of Gly427 by Ala323, so the oxidation occurs at the deepest region of the ligand, for which the double bond is located closer to the iron. Docking performed with HTrA and EPA have not detected good results, while DHA and octacosaoctaenoic acid have shown a good capability to bind the active site, suggesting a preference for a bigger ligand. In both cases, the tails reach the deepest portion of the pocket, while the polar heads are located outside and bind the iron with their C9. From these results, a possible rule as LOX11 could be detected.

For what concerns hypothetical LOX and *Chaetoceros debilis*, docking results highlighted good binding affinity with EPA. The ligand binds the active site with its tail oriented in the deepest portion of the pocket and its polar head outside. The carbon that interacts with the iron is the C7 and the oxidation takes place at the carbon 9, due to the closure of the O2 upper site by the Ala385l; thus, this LOX could be classified as LOX9. Gly537 and Gly556, useful for a deep location of the substrate tail, replace the deepest portion of the pocket flanked by Ala589/620. All the residues involved in the correct position of the ligand are preserved except Ile626 and Leu 627, which are replaced by Thr562/563, probably responsible of the different position of the iron attack despite the one seen for EPA in its interaction with other LOX as the LOX from *P. arenysensis*.

## 4. Discussion

### Putative LOX Sequences Detection, Analysis and Classification

As mentioned at the top of the results section, at the beginning of the work presented, the only sequences annotated as diatoms LOX in databases were the ones from *P. arenysensis*, *F. cylindrus*, and *N. inconspicua*; the latter was wrongly annotated, as demonstrated by our analysis and modelling. However, it is correct to mention that since August 2023, LOX from *Skeletonema marinoi* has been annotated as lipoxygenase too. However, even if at the beginning of the present study this annotation was not available, we still considered LOX from *S. marinoi* in the analysis due to its similarity with the LOX from *P. arenysensis* (35% of sequence identity on 70% of query coverage) and its difference from the LOX of *F*. *cylindrus*. In fact, it became one of the reference structures chosen for representing its relative diatoms group. Therefore, this novel annotation is a further element that corroborates our sequence detection and increases our confidence about the effective LOX nature of the sequence clustered with it.

For what concerns the LOX sequences from *F. cylindrus* and *P. arenysensis*, it is important to underline that from their analysis, it was possible to notice that although both conserved the LOX domain, these two sequences are very different from each other, as different is their cofactor coordination site. Generally, LOX domains present a coordination site composed of three histidine residues, one asparagine, and one leucine or isoleucine, organized along the sequence in a specific way. In particular, the first two histidine residues are often located in the first half of the LOX domain sequence, spaced by four amino acids, while the second histidine, followed by the asparagine after three residues, is located in the second half of the domain; leucine/isoleucine instead is positioned at the end of the sequence.

The LOX from *F. cylindrus* and the similar sequences (groups 4 and 5) present an insertion between the third histidine and the asparagine, which are spaced out by four amino acidic residues. For what concerns LOX from *F. cylindrus*, the tricky point was represented by the presence of an asparagine residue after the third histidine of coordination that might have suggested a possible role of this residue in the coordination. However, the model of the LOX from *F. cylindrus* created displays that are possibly a rearrangement of the helix forming the active site that hosts these residues in a manner to correctly orient the histidine and the asparagine residues near the iron ion, despite the presence of other residues between them. AlphaFold model (AlphaFold DB code: A0A1E7FK81) for this protein sequence has the disadvantage of presenting the coordination residues in an incorrect orientation, determining the presence of the inserted Ala619 in position for the iron coordination. However, on the other hand, considering the nature of this AI, it could not be otherwise, due to the absence of structures characterized by this insertion in the active site among the ones stored in the PDB database and the absence of the cofactor in the model released. Therefore, it was necessary to exploit other approaches for the building of a model in an active conformation, functional for docking analyses, and that could be a reference for the modelling of similar sequences.

Docking results have shown that different residues in the active site pocket result in different LOX types making different interactions with the substrates. In particular, the similar composition of the active site between LOX from *P. homomalla* and *P. arenysesnis* determines the attack on the same carbon atom (C10) for substrates with the same length. The difference in terms of LOX products is attributable to the different composition of the oxygen channel that allows the oxidation in the deepest portion of the substrate (C12). The result obtained is also in agreement with the literature data, which report a possible role of LOX from *P. arenysensis* as LOX12 or 15, due to the discovery of oxylipins produced by this diatom by stereo- and regio-selective oxidation of EPA at C12 and C15 [47]. An example of this is also given by the hypothetical LOX from *P. tricornutum*, for which, as explained in the result section, the active site presents many differences that can be related to the absence of interaction with EPA and the possible binding with the 9,12-hexa-decadienoic acid. The preference for this substrate, despite the EPA, could be related to the major cellular availability of 9,12-hexa-decadienoic acid by these kinds of diatoms, which produce in principle polyunsaturated fatty acid with 16-carbon atoms [48], and that could have driven the LOX active site for this organism in a composition more suitable for this kind of PUFA.

Similarly, docking analyses highlight that a crucial role in the substrate preference is also played by the composition of the deepest portion of the catalytic pocket. *S. marinoi* LOX, for example, while still preserving the composition of the catalytic area, shows a reduction in the deepest pocket part, that does not allow the substrates to reach deeper into the pocket. This results in a different attack of EPA on the binding side, in a different carbon atom that can interact with the substrate, and in a binding capability, as highlighted by the quite comparable binding energy detected, also for smaller substrates as HTrA. These results are in line with a literature study reporting the possible presence of an LOX9 pathway acting on HtrA in *Skeletonema marinoi* [49].

The impact of the pocket shape and composition on the method of binding the substrates is even more visible in that LOXs that present an insertion among their coordination residues. The slight change in the active site shape, due to the presence of another amino acidic residue, and the difference in terms of residues forming the whole catalytic pocket seem to lead to a different adaptability to different substrates. In LOX from *F. cylindrus*, for example, as demonstrated by the comparable binding energies between the two poses, these changes lead to a different binding conformation of EPA that could result in the generation of different products. In a similar way, LOX from *A. glacialis* seems to be adaptable to different kinds of substrates, even if the lowest binding energy values and the Ki seem to decrease as the length of the substrates increases. The enzyme seems to prefer bigger substrates; however, the difference seems to be imputable not to the pocket depth that results still comparable in this area to the ones detected for LOX from *P. arenysensis* and *S. marinoi*, but to the different compositions of the residues located in the middle of the cavity responsible for the substrate conformation. The pocket shape conformational change seems to also affect LOX from *Bellerochea* sp. that, because of a cavity that is tighter in the central part but wider in the deepest portion, seems to bind bigger ligands better than the smaller ones. LOX from *C. debilis*, instead, despite the insertion, is the one that seems to have the most borderline behavior; the helix break is smaller and there is an inferior conformational change in the pocket that remains in shape quite similar to the one of LOX from *P. arenysensis*, for example. In this case, what changes are some residues in the pocket that again may become crucial for the substrate to bind the pocket. Despite the good binding affinity detected for EPA, consistent with the literature studies [50] that highlighted these diatoms as a considerable producer of this fatty acid, it is probable that products generated from these substrates are typical of a LOX9.

From our results noteworthy are also the LOX type detected for LOX from *P. dubia.* This enzyme seems to reveal a possible preference for bigger substrates such as the DHA and the octacosa-octaenoic acid, a substrate for which this LOX seems to assume the role of a LOX 7 or LOX16, respectively. LOX 7 and LOX16 appear rare but not absent in nature; for example, LOX7 is present in *Z. mays*, while LOX16 has been recently discovered in *Cucumis sativus* L. [51].

## 5. Conclusions

Diatom lipoxygenase evolution reflects the dynamic interplay between these microorganisms and their environment. Over millions of years, diatoms have fine-tuned their lipoxygenase enzymes to cope with diverse ecological challenges. Evolutionary pressures have driven the development of specific lipoxygenase isoforms tailored to respond to environmental cues, such as nutrient availability and predation threats. The evolution of diatom lipoxygenase has played a pivotal role in shaping the algae’s resilience, adaptability, and ecological success. Understanding the molecular evolution of these enzymes provides insights into the intricate mechanisms that allow diatoms to thrive in a wide range of aquatic habitats, contributing to the broader understanding of evolutionary processes in microbial ecosystems. In this context, the description of molecular aspects on the substrate selection may open the way to further experimental analyses for the selection of oxylipins of biotechnological interest.

## Figures and Tables

**Table 1:** Reference sequences and their templates. The table reports information related to the NCBI accession number of the putative LOX sequences, their annotation, the organism of origin, and the two templates used for modelling performed by Modeller10.2.

NCBI Accession Number	Annotation	Organism	Template 1	Template 2	Sequence Identity
OEU18547.1	lipoxygenase	*Fragilariopsis cylindrus*	SWISS-MODEL model	3FG1	20.88%
QWC64745.1	lipoxygenase	*Pseudo-nitzschia arenysensis*	SWISS-MODEL model	1HU9	26.64%
CAD9618313.1	unnamed protein product	*Skeletonema marinoi*	AlphaFold model	LOX from *P. arenysensis* model	45.32%
XP_002180840.1	predicted protein	*Phaeodactylum tricornutum*	AlphaFold model	LOX from *P. arenysensis* model	32.10%
CAD8595700.1	unnamed protein product	*Asterionellopsis glacialis*	AlphaFold model	LOX from *F. cylindrus* model	26.22%
GJWO01009215.1	transcribed RNA sequence	*Bellerochea* sp.	AlphaFold model	LOX from *F. cylindrus* model	22.62%
CAE0460244.1	unnamed protein product	*Chaetoceros debilis*	AlphaFold model	LOX from *F. cylindrus* model	23.61%
CAD8307630.1	unnamed protein product	*Pseudictyota dubia*	AlphaFold model	LOX from *P. arenysensis* model	22.30%

**Table 2:** Most relevant docking results. The binding energy column indicates the medium binding energy calculated from the mean of all the binding energies detected for each binding pose belonging to the cluster in exam. The acronym l.b.e reported in round brackets stands for lowest binding energy and is related to the pose in the cluster that assumes the lowest energy of interaction.

Group	Organism of Origin	Substrate	Binding Energy (Kcal/mol)	N° of Poses in Cluster	Ki (µM)	Residues of Interaction (H-bonds are underlined)	LOX Type
control	*Plexaura homomalla*	AA	−6.95 (l.b.e.= −7.40)	15	3.74	Tyr178-Arg182-Gln380-His384-Leu385-His389-Gly427-Leu431-Ser441-Leu444-Thr623-Val624-Leu627-Fe^2+^-H_2_O	LOX8
1	*Skeletonema marinoi*	EPA	−5.46 (l.b.e.= −7.04)	11	6.93	Gln266-Trp267-His270-Leu271-His275-Ser317-Leu318-Phe326-Gln330-Met529-Leu533-Ile599	LOX9
		HTrA	−5.52 (l.b.e.= −5.79)	11	56.56	Ala213-Gln266-Leu271-His275-Ala314-Leu318-Phe326-Gln330-Ser526-Met529-Val530-Leu533	LOX9
		HTrA	−4.89 (l.b.e.= −5.46)	4	334.45	Gln266-His270-Leu271-His275-Ala313-Ala314-Ser317-Leu318-Phe326-Gln330-Met529-Leu533	LOX9
2	*Pseudo-nitzschia arenysensis*	EPA	−7.59 (l.b.e.= −7.59)	1	2.75	Glu388-Trp389-Leu393-Leu432-Thr439-Leu440-Phe448-Gln583-Ile634-Ile637-Leu638-Ile704-Fe^2+^-H_2_O	LOX12
3	*Phaeodactylum tricornutum*	9,12-hexa-decadienoic acid	−5.62	1	75.75	Ala97-His101-His105-Ser102-Phe352-Ala353-Phe356-Val357-Ala147-Val150-Gln151-Phe198- Trp418-Fe^2+^	LOX12
4a	*Fragilariopsis cylindrus*	EPA	−5.63 (l.b.e.= −5.81)	2	54.97	Phe160-His164-Leu165-Ile203-Ala207-Leu211-Ala355-Asn356-Val397-Leu400-Thr401-Fe^2+^-H_2_O	LOX8
			−5.51	1	91.52	Phe160-His164-Leu165-His169-Ile203-Ala207-Leu211-Ile218-Thr221-Val397-Thr401-Leu478-Fe^2+^	LOX15
4b	*Asterionellopsis glacialis*	EPA	−5.25	1	141.17	Thr459-His463-Leu464-Val503-Ser506-Ala507-Ile510-Leu511-Phe517-Ile518-Trp522-Leu663-Leu690-Thr697-Fe^2+^	LOX9
		DHA	−4.77 (l.b.e.= −5.60)	4	78.95	His463-Leu464-Leu511-Phe517-Ile518-Trp522-Leu690-Leu693-Val694-Ser696-Thr697-Leu77-Fe^2+^	LOX14
		Octacosa-octaenoic acid	−6.50 (l.b.e.= −6.93)	3	17.41	Phe-455-Thr459-His463-Leu464-Val503-Ser506-Ala507-Leu511-Phe517-Ile518-Val521-Trp522-Leu690-Leu693-Val694-Thr697-Ser766-Leu767	LOX20
5a	*Bellerochea* sp.	DHA	−5.20 (l.b.e.= −6.29)	6	25.02	His279-Thr275-Leu280-Thr322-Ala323-Ala326-Leu327-Leu333-Phe337-Glu461-Thr471-Ser488-Leu491-Met492-Leu494-Val558-H_2_O	LOX11
		Octacosa-octaenoic acid	−5.80 (l.b.e.= −7.51)	6	3.14	Val271-Thr275-Ile319-Thr322-Ala323-Leu327-Leu333-Glu461-Thr464-Ser465-Ala469-Gly470-Trp485-Ser488-Leu491-Met492-Leu494-Val558-Fe^2+^-H_2_O	LOX11
5b	*Chaetoceros debilis*	EPA	−5.78 (l.b.e.= −5.87)	6	49.54	Thr337-His341-Leu342-His346-Ser388-Leu389-Thr395-Ile396-Ala399-Phe538-Ile559-Thr562-Thr563-Fe^2+^-H_2_O	LOX9
6	*Pseudictyota dubia*	EPA	−5.64 (l.b.e.= −5.89)	9	47.91	Gln256-Met257-Phe260-His264-Asn298-Gly302-Leu306-Tyr313-Phe482-Leu483-Phe486-Phe487-H_2_O	LOX8
		DHA	−6.55 (l.b.e.= −7.35)	6	4.08	Phe82-Gln256-Phe260-His264-Asn298-Ala301-Gly302-Leu306-Tyr313-Trp317-Gly439-Ser443-Ile444-Leu483-Thr484-Phe487	LOX4/7
		Octacosa-octaenoic acid	−6.53 (l.b.e.= −7.97)	19	1.43	Phe82-Gln256-Met257-Phe260-His264-His259-Asn298-Ala301-Gly305-Leu306-Thr311-Thr312-Tyr313-Trp317-Leu483-Phe487	LOX16

## Data Availability

The data used in this study were available in resources available in the public domain (listed under the Section 2). The original contributions presented in the study are included in the article/Supplementary Material, further inquiries can be directed to the corresponding author.

## Supplementary Materials

The following supporting information can be downloaded at: https://www.mdpi.com/article/10.3390/biom14030276/s1, Figure S1: Visualization of the “U-shaped” pocket of the LOX from *P. homomalla* and details of the interactions between substrate and cofactors; Figure S2: Motif research along the sequences belonging to groups 1 and 2.; Figure S3: 3D structural conformation of the cofactor coordination residues present in the structure of LOXs; Table S1: Grid parameters for docking simulations; Table S2: List of the 45 sequences found to be similar to LOXs from *P. arenysensis* or *F. Cylindrus*.
