# Supplementary material for: Structural and Functional Characterization of Lipoxygenases from Diatoms by Bioinformatics and Modelling Studies
Source: Biomolecules. 2024 Feb 25;14(3):276. doi: 10.3390/biom14030276 (PMC10968607; doi:10.3390/biom14030276)
Supplement: Supplementary file 1 [file biomolecules-14-00276-s001.zip › Supplementary Figure_S1.pdf]

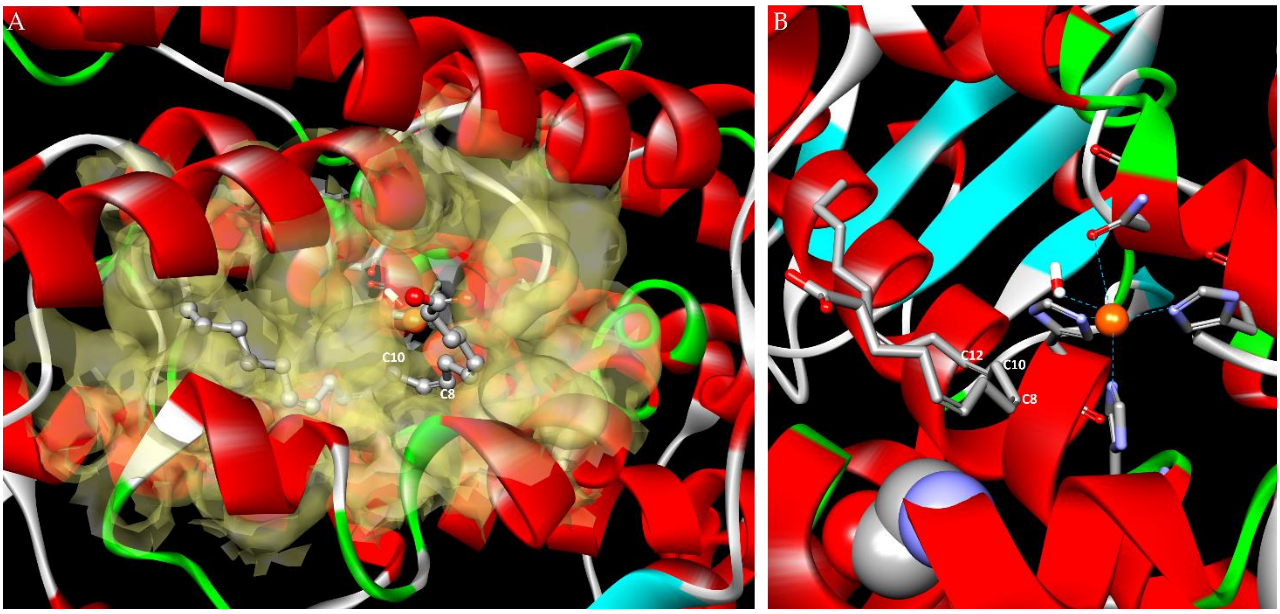

Supplementary Figure S1: visualization of the “U-shaped” pocket of the LOX from *P. homomalla* (PDB code: 4QWT) and of the interactions between substrate (arachidonic acid) and cofactors (Iron and water). In panel A: the substrate, located with its polar head outside the pocket, makes interaction with iron cofactor by its C10, while double bond of C8 can be reached by oxygen molecules due to the presence of a Gly residue in position 427 that allows its passage. In panel B: detailed view of the cofactor, located at the center of the “U pocket”, and its coordination residues. The iron cofactor extracts a hydrogen atom to the nearest carbon of the substrate, at two positions upstream the oxidation of the double bond if the polar head is located outside the pocket (C8 in this case), downstream if the substrate polar head is oriented inside (C12 if the arachidonic acid was bound in an upside-down conformation). In this panel, protein is visualized in cartoon (colored by secondary structure), Gly427 is in cpk, coordination residues, substrate, and water molecule in stick, while the iron ion is in orange sphere.
