# Supplementary material for: Structural and Functional Characterization of Lipoxygenases from Diatoms by Bioinformatics and Modelling Studies
Source: Biomolecules. 2024 Feb 25;14(3):276. doi: 10.3390/biom14030276 (PMC10968607; doi:10.3390/biom14030276)
Supplement: Supplementary file 1 [file biomolecules-14-00276-s001.zip › Supplementary Figure_S2.pdf]

A Discovered Motifs

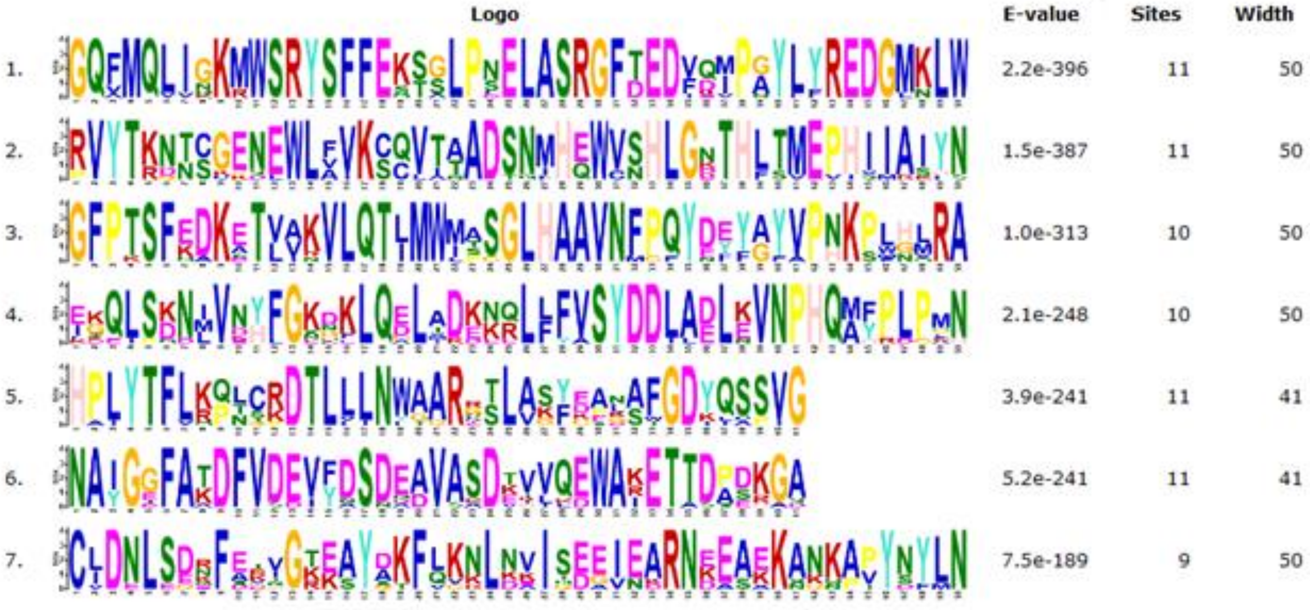

B

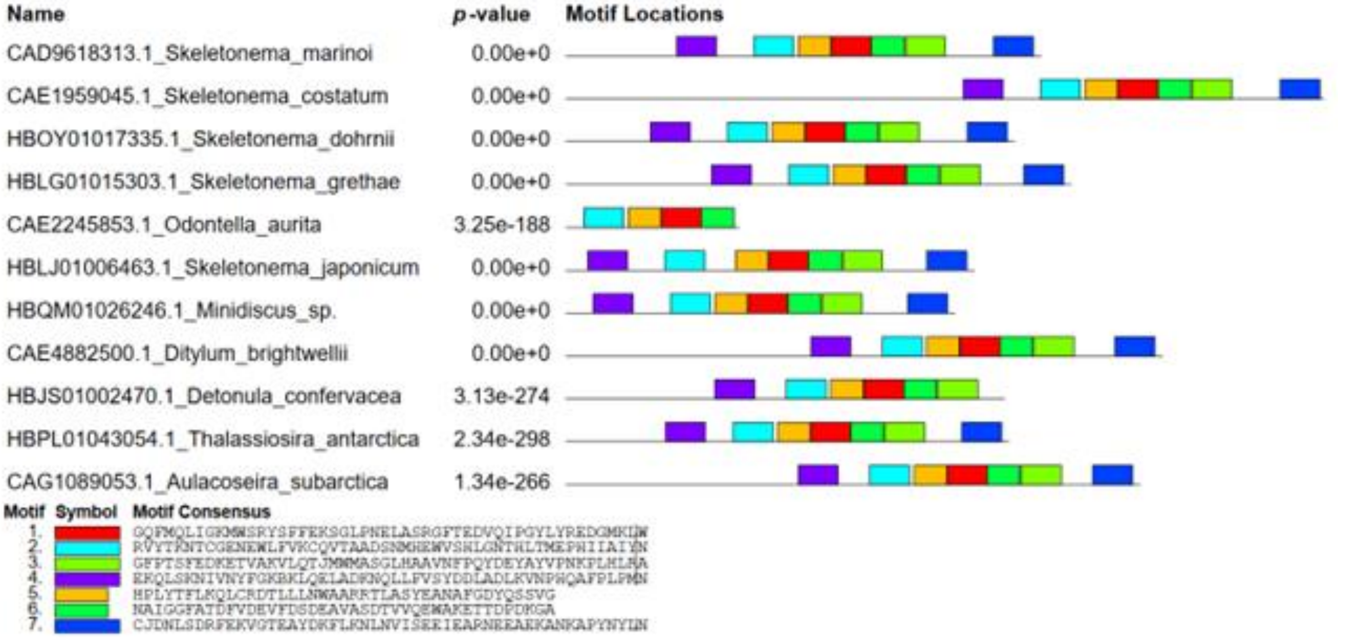

C Discovered Motifs

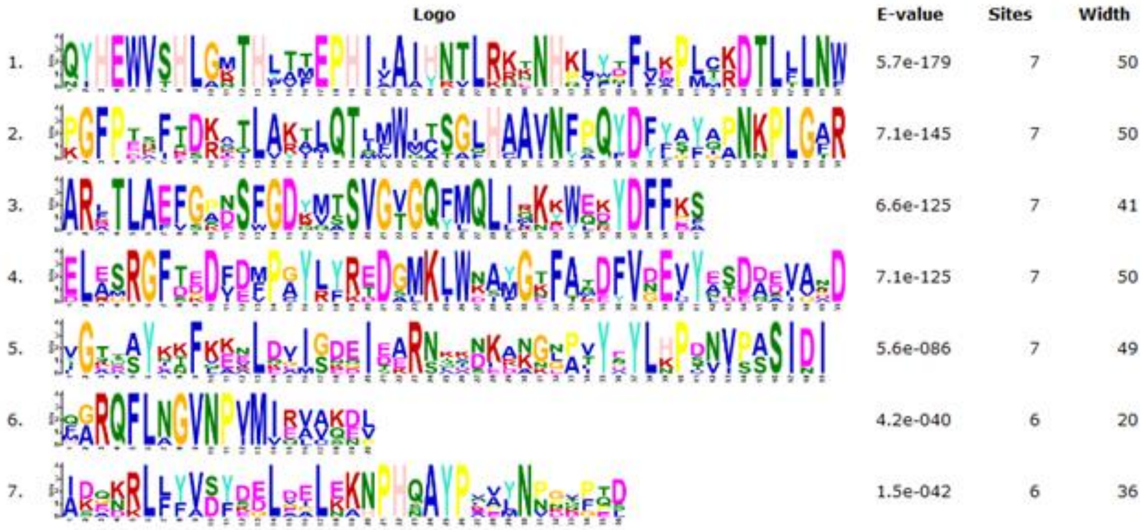

D

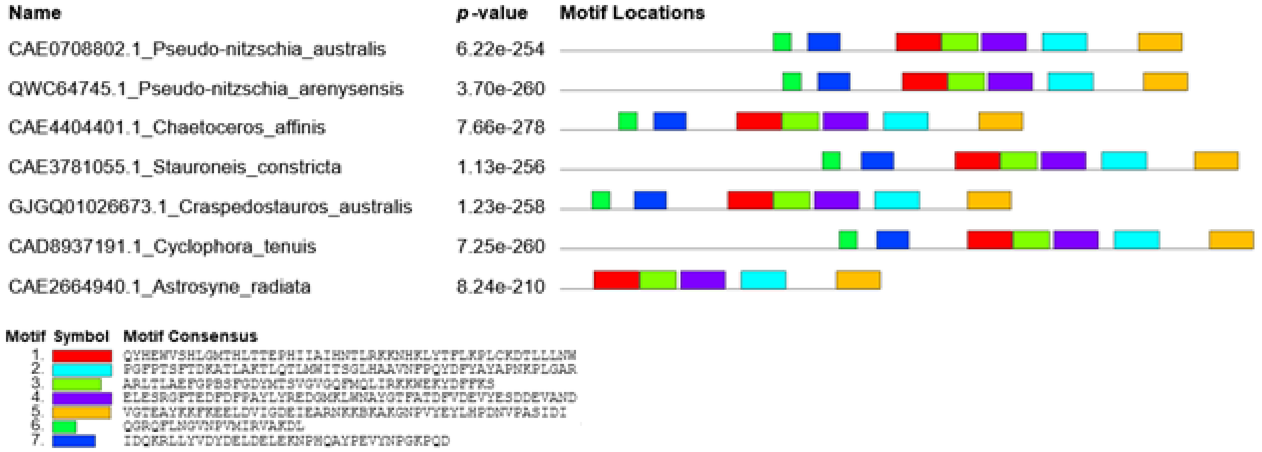

E

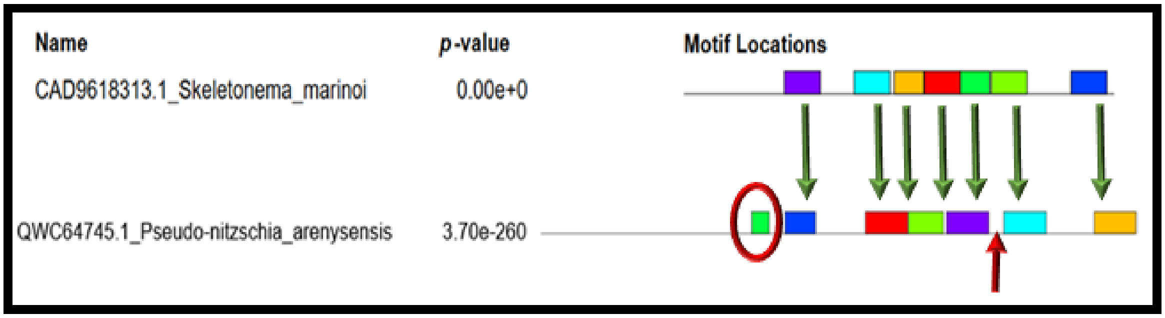

Supplementary Figure S2. Motif research along the sequences belonging to groups 1 and 2. (A) Motif discovered in group 1; (B) motif locations along each sequence analyzed for group 1 (C) motif discovered in group 2; (D) motif locations along each sequence analyzed for group 2; (E) motif comparison between the two groups; green arrows indicate the correspondence between two motif detected, while the red arrow points the attention on the last portion of motif 3 and the first portion of motif 6 present in group 1, that are not detectable in group 2. Similarly, the red circle around motif six detectable at the beginning of all sequences belonging to group 2 highlights as this motif is missing in group 1 sequences.
