# Supplementary material for: Structural and Functional Characterization of Lipoxygenases from Diatoms by Bioinformatics and Modelling Studies
Source: Biomolecules. 2024 Feb 25;14(3):276. doi: 10.3390/biom14030276 (PMC10968607; doi:10.3390/biom14030276)
Supplement: Supplementary file 1 [file biomolecules-14-00276-s001.zip › Supplementary Figure_S3.pdf]

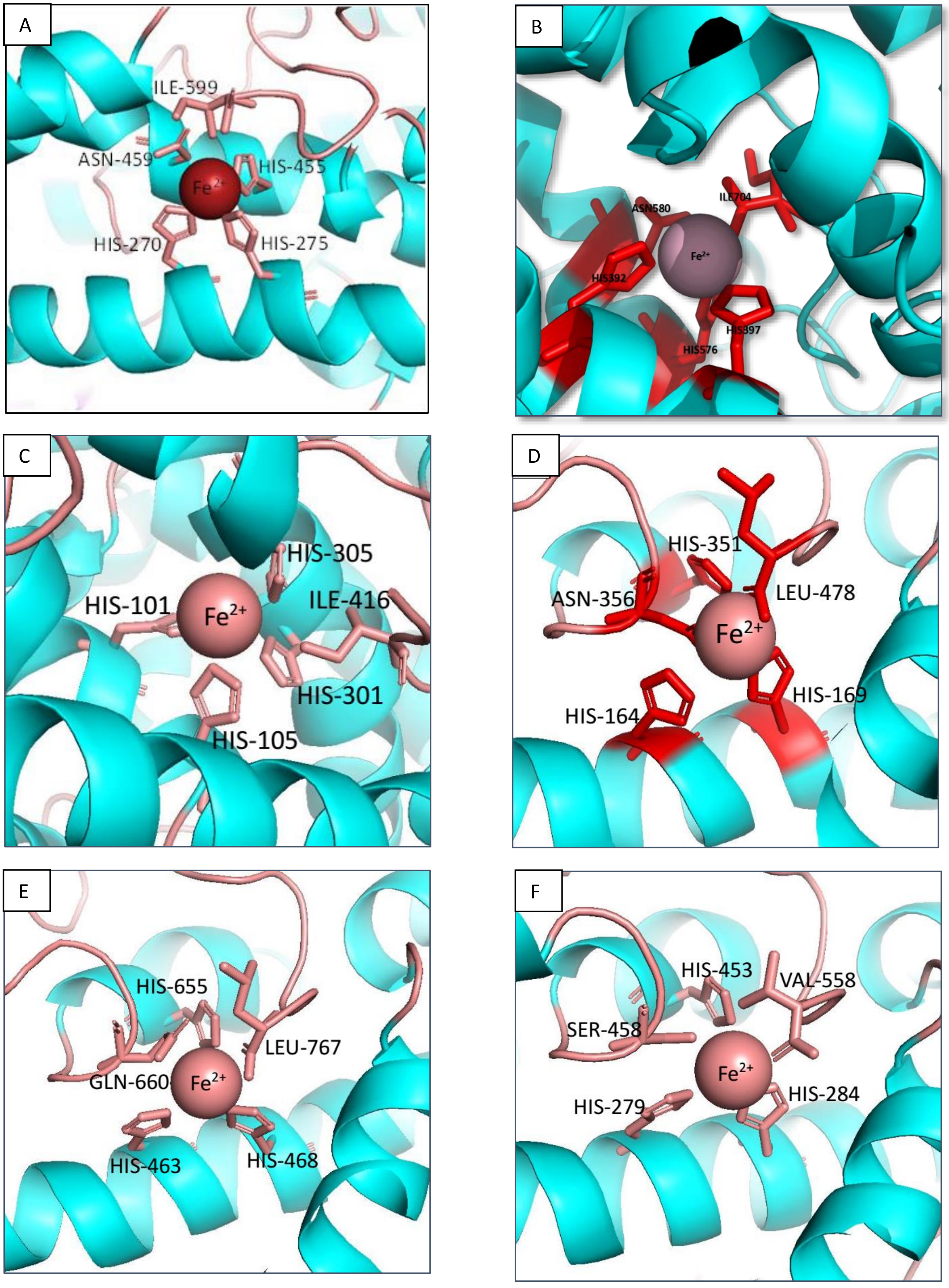
A
ILE-599
ASN-459
HIS-455
Fe2+
HIS-270
HIS-275
B
ASN580
ILE704
Fe2+
HIS392
HIS397
HIS576
C
HIS-305
HIS-101
Fe2+
ILE-416
HIS-301
HIS-105
D
HIS-351
ASN-356
LEU-478
Fe2+
HIS-169
HIS-164
E
HIS-655
LEU-767
GLN-660
Fe2+
HIS-463
HIS-468
F
HIS-453
VAL-558
SER-458
Fe2+
HIS-284
HIS-279

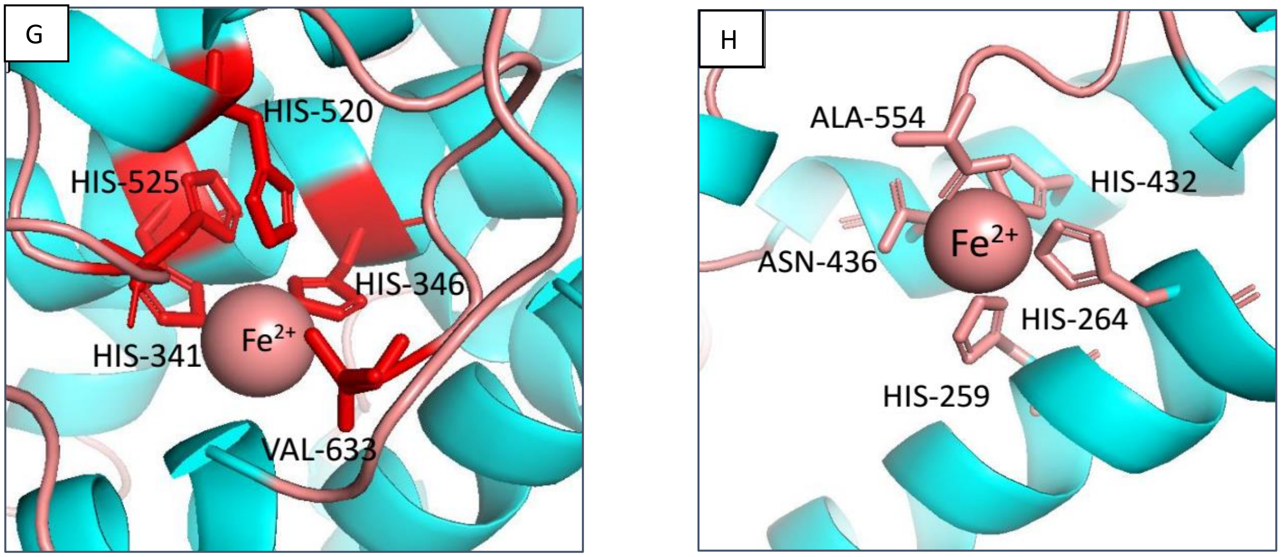

Supplementary Figure S3. 3D structural conformation of the cofactor coordination residues present in the structure of LOXs from *S. marinoi* (A), *P. arenysensis* (B), *P. tricornutu* (C), *F. cylindrus* (D), A*. glacialis* (E), *Bellerochea sp*. (F), *C. debilis* (G), and from *P. dubia* (H).
