# Supplementary material for: Structural and Functional Characterization of Lipoxygenases from Diatoms by Bioinformatics and Modelling Studies
Source: Biomolecules. 2024 Feb 25;14(3):276. doi: 10.3390/biom14030276 (PMC10968607; doi:10.3390/biom14030276)
Supplement: Supplementary file 1 [file biomolecules-14-00276-s001.zip › Supplementary Table_S1.pdf]

Supplementary Table S1. Docking simulations summary and grid parameters used.

The first line refers to LOX from *P. homomalla* , separated to highlight that this LOX is not from diatom organism, and has been used as a reference. This is the protein present in PDB database with a structure complexed to arachidonic acid (AA). Our docking simulations for this protein were aimed to check the reliability of the docking procedure. The results evidenced high affinity, and the perfect reproduction of the binding mode.
The other 8 LOX structures are from diatoms. We performed docking simulations on these structures to investigate their ability to bind different substrates and propose their preliminary classification.
The second LOX we presents in the table is from *P. tricornutum* . In this case, from the literature it is suggested that the substrate is 9,12-hexa-decadienoic acid. We performed docking simulations with this substrate and, in addition, with EPA, to verify that the docking simulations are able to differentiate correct vs incorrect substrates. The results confirm the better affinity of 9,12-hexa-decadienoic acid instead of EPA. Moreover, the correct position in the binding site is possible only for 9,12-hexa-decadienoic acid (not shown).
The table continues with the results related to *A. glacialis, Bellerochea sp., P. dubia* for which no evidence in literature suggests the possible substrate. We performed the simulations with four substrates having different length (HTrA, EPA, DHA, octacosaoctaenoic acid), to evaluate the substrate preference and obtain a comparison among the different lengths.
Finally, the table reports the results for the other diatom LOXs. In these cases, we performed the docking analysis only with the substrates suggested by literature evidences, for a further comparison to the energy value of our reference complex (i.e., *P. homomalla*).
The column with the header “Confirmation of binding” (for *P. homomalla*) or “Potential binding” reports YES/NO to indicate that the docking simulations suggest or not the binding of the ligand. “Higher affinity” is reported when the best docking score obtained was comparable to the one detected for our reference complex.

	Docking results		Docking settings							
	**Substrate**	**Confirmation of binding**	**npts grid points in xyz**			**spacing(A)**	**gridcenter xyz-coordinates or auto**			**flexible residues**
Redocking check										
**LOX from *P. homomalla***	**arachidonic acid(AA)**	**YES (HIGH AFFINITY)**	94	72	82	0.375	5.343	-2.223	6.921	null
	**Substrate**	**Potential binding**	**npts grid points in xyz**			**spacing(A)**	**gridcenter xyz-coordinates or auto**			**flexible residues**
Diatom LOXs docking analysis										
**LOX from *P. tricornutum***	**EPA**	**NO**	56	50	58	0.375	9.339	-1.971	6.24	null
	**9,12-hexa-decadienoic acid ***	**YES**	60	50	60	0.375	10.183	-1.92	6.172	H-105, F-352, F-356, W-418
**LOX from *A. glacialis***	**HTrA**	**NO**	64	50	60	0.375	5.503	-0.564	11.792	F-517, I-518, W-522, L-690, L-693
	**EPA**	**YES**	64	50	60	0.375	5.456	-0.579	11.771	F-517, I-518, W-522, L-690, L-693
	**DHA**	**YES**	64	50	60	0.375	5.474	-0.312	11.904	F-517, I-518, W-522, L-690, L-694
	**octacosaoctaenoic acid**	**YES (HIGHER AFFINITY)**	64	50	60	0.375	6.716	1.248	11.918	F-517, I-518, W-522, L-690, L-695
**LOX from *Bellerochea sp.***	**HTrA**	**NO**	64	50	60	0.375	-7.677	0.9	-0.675	L-333, L-491, L-494
	**EPA**	**NO**	64	50	60	0.375	-7.331	1.056	-0.643	null
	**DHA**	**YES**	64	50	60	0.375	-7.677	0.9	-0.675	L-333, L-491, L-494
	**octacosaoctaenoic acid**	**YES**	64	50	60	0.375	-7.677	0.9	-0.675	L-333, L-491, L-495
**LOX from *P. dubia***	**HTrA**	**NO**	64	50	60	0,375	8,918	2,408	2,847	null
	**EPA**	**YES**	64	50	60	0.375	7.28	2.704	2.854	null
	**DHA**	**YES (HIGHER AFFINITY)**	64	50	60	0.375	8.918	2.408	2.847	null
	**octacosaoctaenoic acid**	**YES (HIGHER AFFINITY)**	64	50	60	0.375	7.263	2.352	2.819	null
**LOX from *S. marinoi***	**HTrA ***	**YES**	56	50	58	0.375	-8.969	4.555	-8.19	null
	**HTtA ***	**YES**	56	50	58	0.375	-12.055	4.444	-7.437	null
	**EPA ***	**YES (HIGHER AFFINITY)**	56	50	58	0.375	-6.961	4.556	-8.189	null
**LOX from *P. arenysensis***	**EPA ***	**YES (HIGHER AFFINITY)**	78	82	70	0.375	18.497	2.754	15.254	null
**LOX from *F. cylindrus***	**EPA ***	**YES**	78	82	70	0.375	7.849	-1.152	-0.932	null
**LOX from *C. debilis***	**EPA ***	**YES**	56	50	60	0.375	-8.056	-1.546	-2.962	null

* substrate whose production in the specific diatom species is suggested by literature
