# Supplementary material for: Structural and Functional Characterization of Lipoxygenases from Diatoms by Bioinformatics and Modelling Studies
Source: Biomolecules. 2024 Feb 25;14(3):276. doi: 10.3390/biom14030276 (PMC10968607; doi:10.3390/biom14030276)
Supplement: Supplementary file 1 [file biomolecules-14-00276-s001.zip › Supplementary Table_S2.pdf]

Supplementary Table S2. List of the 45 sequences found to be similar to LOXs from *P. arenysensis* or *F. Cylindrus.* Sequences have been divided in six groups according to their cofactor coordination residues pattern and their placement in the phylogenetic tree (Figure 1 in the manuscript). Particularly, the same background color presents in each line of the same group corresponds to the clustering of these sequences under the same branch in the tree; color highlighting each NCBI Entry code corresponds to the color label present for these sequences in the tree, that is related to a similar residues' composition of the iron coordination pattern.

Entry NCBI	Entry UniProt	Organism of origin	Sequence type	Sequence length	Iron coordination pattern
**GROUP I**					
CAD9618313	A0A7S2LXV6	*Skeletonema marinoi*	protein	599	HlgktH – HaavN
CAE1959045		*Skeletonema costatum*	protein	955	HlgktH – HaavN
HBOY01017335		*Skeletonema dohrnii*	RNA	566	HlgntH – HaavN
HBLG01015303		*Skeletonema grethae*	RNA	637	HlgntH – HaavN
CAE2245853	A0A7S4J0G1	*Odontella aurita*	protein	219	HlgntH
HBLJ01006463		*Skeletonema japonicum*	RNA	515	HlgntH – HaavN
HBQM01026246		*Minidiscus sp.*	RNA	491	HlgetH – HaavN
CAE4882500		*Ditylum brightwellii*	protein	752	HlgntH – HaavN
HBJS01002470		*Detonula confervacea*	RNA	553	HlgdtH – HaavN
HBPL01043054		*Thalassiosira antarctica*	RNA	559	HlgdtH – HaavN
CAG1089053		*Aulacoseira subarctica*	protein	724	HlgrtH – HaavN
**GROUP II**					
HBGY01034511		*Leptocylindrus danicus*	RNA	612	HlantH – HagvN
CAE0708802	A0A7S4EF52	*Pseudo-nitzschia australis*	protein	698	HlgmtH – HaaiN
QWC64745	A0A8E8LBZ9	*Pseudo-nitzschia arenysensis*	protein	704	HlgmtH – HaavN
CAE4404401		*Chaetoceros affinis*	protein	519	HlamtH – HcavN

CAE3781055		*Stauroneis constricta*	protein	761	HlgktH – HaavN
GJGQ01026673		*Craspedostauros australis*	RNA	506	HlgktH – HaavN
CAD8937191	A0A7S1D4P1	*Cyclophora tenuis*	protein	778	HlgntH – HaavN
CAE2664940		*Astrosyne radiata*	protein	359	HlgrtH – HaavN
CAE5663449		*Fragilariopsis kerguelensis*	protein	712	HlaktH – HaavN
**GROUP III**					
XP_002180840	B7G1E2	*Phaeodactylum tricornutum*	protein	596	HswvH – HstdH
HBME01023157		*Synedropsis cf. recta*	RNA	588	HswvH – HstdH
CAD8884873	A0A7S1BET1	*Corethron hystrix*	protein	569	HatiH and Hgatl
**GROUP IV**					
CAD9818490	A0A7S2UHJ2	*Attheya septentrionalis*	protein	1378	HlvnvH – HelvgQ
GHBX01011490		*Nitzschia palea*	RNA	448	HlvnvH – HelvgQ
CAD8595700	A0A7S0KYY3	*Asterionellopsis glacialis*	protein	767	HlghcH – HehvgQ
OEU18547	A0A1E7FK81	*Fragilariopsis cylindrus*	protein	742	HlleiH – HnlvaN
HBQV01033768		*Synedra*	RNA	716	HllevH – HnvvaN
HBSF01004517		*uncultured Nitzschia*	RNA	360	HlleiH – HnlvaN
CAE3758544		*Chaetoceros cf. neogracilis*	protein	735	HlsveH – HssigN
**GROUP V**					
GJWO01009215		*Bellerochea sp.*	RNA	558	HlfnaH – HehvgS
HBQT01021118		*Navicula sp.*	RNA	511	HlynlH – HeqvgS
HBRE01028634		*Pleurosigma sp.*	RNA	585	HllwcH – HdlvgS
CAE4385921		*Chaetoceros affinis*	protein	555	HlvwsH – HelvgA
HBOX01063848		*Proboscia alata*	RNA	306	HlvwsH – HeimgH
HBPD01019733		*Thalassionema nitzschioides*	RNA	582	HliwtH – HelvgH

HBPJ01045426		*Thalassiosira minuscula*	RNA	358	HlcqtH – HelvgH
CAE0460244	A0A7S3V6L4	*Chaetoceros debilis*	protein	633	HlaqtH – HeiigH
GFH44228		*Chaetoceros tenuissimus*	protein	624	HlaqsH – HeligH
CAB9509299		*Seminavis robusta*	protein	576	HltwsH – HelvgN
**GROUP VI**					
CAD8419348	A0A7S0CCN1	*Proboscia inermis*	protein	336	HfvysH – HhswN
CAD8307630	A0A7R9VXA5	*Pseudictyota dubia*	protein	554	HfietH – HhvmN
CAE3007537		*Stephanopyxis turris*	protein	1179	HaltcH – HaavN
CAF33213		*Phaeodactylum tricornutum*	protein	937	HfsrcH – HaavN
